# Transcatheter Aortic Valve Replacement for Failed Surgical or Transcatheter Bioprosthetic Valves: A Comprehensive Review

**DOI:** 10.3390/jcm13051297

**Published:** 2024-02-25

**Authors:** Taylor Groginski, Amr Mansour, Diaa Kamal, Marwan Saad

**Affiliations:** 1 Department of Medicine, The Warren Alpert Medical School of Brown University, Providence, RI 02912, USA; tgroginski@lifespan.org; 2 Department of Cardiology, Ain Shams University, Cairo 11566, Egypt; amrmansour@med.asu.edu.eg (A.M.); drdiaaeldin@med.asu.edu.eg (D.K.); 3 Department of Medicine, Division of Cardiology, The Warren Alpert Medical School of Brown University, Providence, RI 02912, USA

**Keywords:** TAVR, valve in valve, SAVR, review, outcomes, aortic stenosis, prosthesis–patient mismatch, paravalvular regurgitation, coronary obstruction

## Abstract

Transcatheter aortic valve replacement (TAVR) has proven to be a safe, effective, and less invasive approach to aortic valve replacement in patients with aortic stenosis. In patients who underwent prior aortic valve replacement, transcatheter and surgical bioprosthetic valve dysfunction may occur as a result of structural deterioration or nonstructural causes such as prosthesis–patient mismatch (PPM) and paravalvular regurgitation. Valve-in-Valve (ViV) TAVR is a procedure that is being increasingly utilized for the replacement of failed transcatheter or surgical bioprosthetic aortic valves. Data regarding long-term outcomes are limited due to the recency of the procedure’s approval, but available data regarding the short- and long-term outcomes of ViV TAVR are promising. Studies have shown a reduction in perioperative and 30-day mortality with ViV TAVR procedures compared to redo surgical repair of failed bioprosthetic aortic valves, but 1-year and 5-year mortality rates are more controversial and lack sufficient data. Despite the reduction in 30-day mortality, PPM and rates of coronary obstruction are higher in ViV TAVR as compared to both redo surgical valve repair and native TAVR procedures. New transcatheter heart valve designs and new procedural techniques have been developed to reduce the risk of PPM and coronary obstruction. Newer generation valves, new procedural techniques, and increased operator experience with ViV TAVR may improve patient outcomes; however, further studies are needed to better understand the safety, efficacy, and durability of ViV TAVR.

## 1. Background 

Since its introduction in 2002, transcatheter aortic valve replacement (TAVR) has become a safe, effective, and less invasive approach to aortic valve replacement in patients with aortic stenosis (AS), eventually leading to its approval by the Food and Drug Administration (FDA) in 2011 [1,2,3]. TAVR was initially approved for patients deemed to be at high risk or prohibitive risk for surgical aortic valve replacement (SAVR) [4,5]. Since then, randomized controlled trials (RCTs) have shown its safety and efficacy in intermediate-risk patients [6]. More recently, TAVR gained approval for low-risk surgical patients in 2019 after the groundbreaking results of the PARTNER 3 and Evolut-Low Risk trials, showing non-inferiority and possible superiority in comparison to SAVR up to 5 years post-procedure [3,7,8,9,10]. In younger patients with an indication for aortic valve replacement (AVR), the most recent European Society of Cardiology (ESC) and European Association for Cardio-Thoracic Surgery (EACTS) guidelines continue to recommend SAVR over TAVR due to the relative recency of TAVR approval and unknown long-term durability [11]. However, with the indication of TAVR now spanning all surgical risk categories, TAVR has become the most common mode of AVR in patients with AS [11]. The most recent report from the Society of Thoracic Surgeons–American College of Cardiology Transcatheter Valve Therapy (STS-ACC TVT) registry demonstrates an increasing volume of TAVR procedures performed in the United States [2]. In the years 2011–2013 combined, a total of around 13,000 TAVR procedures were performed in the US, and by the year 2019, over 72,000 cases were performed annually [2]. Large increases in procedure volume have also occurred in Europe, as the annual number of TAVR procedures increased from 647 in 2008 to 13,264 in 2014 [12]. Some studies have also shown that as the number of annual procedures increases, both mortality and complication rates have declined [2]. 

Although TAVR is typically performed to replace a native valve, TAVR techniques may also be used to insert a new transcatheter heart valve (THV) in place of a degenerated surgical bioprosthesis (TAV-in-SAV) or THV (TAV-in-TAV) [13,14]. This technique, also known as valve-in-valve (ViV) TAVR, has also been successfully used for the replacement of a THV that had previously replaced a surgical bioprosthetic valve (TAV-in-TAV-in-SAV) [13]. ViV TAVR was first approved in 2015 for patients in whom a redo of a prior transcatheter or surgical bioprosthesis was indicated [3,15]. The most recent American College of Cardiology/American Heart Association (ACC/AHA) guidelines report that ViV TAVR is considered a reasonable (class IIa) treatment for bioprosthetic valve failure in patients with high surgical risk [16]. Redo surgical replacement remains the first recommendation (class I) for patients with low to intermediate risk; however, there are ongoing clinical trials addressing the feasibility, safety, and outcomes of both TAV-in-TAV and TAV-in-SAV in low-risk patients [15,16]. In patients with a failing mechanical valve, TAV-in-SAV is not recommended due to safety risks. To date, one case report in 2021 described successful TAVR implantation for mechanical valve failure in a patient who was deemed to be too high risk for redo surgery [17]. 

With the improvement in surgical bioprosthetic valve durability, the availability of TAV-in-SAV for failed surgical valves, and the advantage of avoiding life-long anticoagulation, a significant shift toward the use of bioprosthetic surgical valves is being observed, even in younger patients [11,18,19]. Both TAV-in-SAV and TAV-in-TAV procedures have become increasingly utilized, with over 4500 ViV TAVR procedures performed in the United States in 2019 [2]. With this recent shift toward surgical bioprosthetic valve replacement, it is expected that the number of TAV-in-SAV procedures could see an even greater increase in the near future. 

### 1.1. Indications for ViV TAVR

#### 1.1.1. Bioprosthetic Valve Dysfunction

Based on the 2020 ACC/AHA guidelines, ViV TAVR carries a class IIa recommendation for high-risk surgical patients with symptomatic aortic valve stenosis or regurgitation as a result of transcatheter or surgical bioprosthetic valve dysfunction [16]. Prosthetic valve dysfunction is most often caused by either the structural degeneration of the valve leaflets or non-structural causes [16,20,21]. Structural valve deterioration is often the result of wear and tear, leaflet disruption, flail leaflet, leaflet fibrosis and/or calcification, or strut deformation that results in permanent damage to the prosthetic valve [20,21,22]. Non-structural valve dysfunction includes any abnormality that is not intrinsic to the function of the valve itself [16,20]. This is often due to paravalvular regurgitation or prosthesis–patient mismatch (PPM), but it may also result from leaflet entrapment, aortic root dilatation, and valve migration/embolization [16,20,21]. Bioprosthetic valve dysfunction may also occur as a result of thrombosis or endocarditis [20]. The most recent Valve Academic Research Consortium (VARC-3) definitions characterize bioprosthetic valve dysfunction by the extent of hemodynamic changes, and the dysfunction is considered as bioprosthetic failure once the patient becomes symptomatic or experiences irreversible hemodynamic valve deterioration (HVD) [20]. Stage 1 HVD is characterized by the presence of structural or non-structural valve deterioration without hemodynamic changes [20]. Stage 2 HVD (moderate HVD) is determined by an increase in mean transvalvular gradient ≥ 10 mmHg resulting in a mean gradient of ≥ 20 mmHg, in addition to a decrease in estimated orifice area (EOA) by ≥ 0.3 cm^2^ (or by ≥ 25%) or a decrease in doppler velocity index by ≥ 0.1 (or ≥ 20%) when compared to an echocardiogram performed 1–3 months post-procedure [20]. Stage 3 HVD (severe HVD) is defined by an increase in mean transvalvular gradient ≥ 20 mmHg resulting in a mean gradient of ≥ 30 mmHg, in addition to a decrease in EOA by ≥ 0.6 cm^2^ (or by ≥ 50%) or a decrease in doppler velocity index by ≥ 0.2 (or ≥ 40%) when compared to an echocardiogram performed 1–3 months post-procedure [20]. Stage 2 and stage 3 HVD may also be characterized by new or worsening intra-prosthetic aortic regurgitation (AR), resulting in moderate and severe AR, respectively [20]. Due to the variability in echocardiographic imaging and assessment, it is recommended that a definitive diagnosis of bioprosthetic valve dysfunction should be based on the results from two serial echocardiograms [20]. 

#### 1.1.2. Paravalvular Regurgitation

Paravalvular regurgitation affects 5–17% of all surgical prosthetic valves and occurs when blood leaks through the space between the native heart and the prosthetic valve [23,24]. This may be a result of annular calcification, eccentricity of the annulus, undersizing of the device, or malpositioning of the valve during deployment [22,25]. A recent meta-analysis of 30 RCTs and observational studies found that 7–40% of THVs resulted in paravalvular regurgitation and 10–25% of cases were considered to be either moderate or severe [25]. For acute paravalvular leaks, repeated post-balloon dilation of an underexpanded valve or the use of a snare loop-assisted device to reposition the valve may minimize significant regurgitation [22,25]. For non-acute paravalvular regurgitation, transcatheter vascular plugs may be utilized to close the paravalvular leak, but these procedures increase the risk of THV embolization and stroke [22,25]. In patients with a surgical bioprosthetic valve that can be fractured, ViV TAVR can be considered, though most patients with low surgical risk will undergo SAVR [22]. In order to reduce the likelihood of paravalvular regurgitation, newer generations of THVs are designed to be repositioned and have a better seal within the native valve annulus [25]. 

#### 1.1.3. Prosthesis–Patient Mismatch

PPM occurs when the implanted THV is too small in relation to the patient’s body size, resulting in a smaller orifice area of the prosthetic valve, increased aortic valve gradients, and diminished cardiac output [23,26,27]. Studies have demonstrated that PPM leads to increased re-admission rates (often for heart failure or redo valve replacement) and significantly reduces long-term survival [11,26]. The severity of prothesis–patient mismatch is determined using the indexed effective orifice area (iEOA). This is calculated by dividing the effective orifice area by the patient’s body surface area [26]. Severe PPM is defined as an iEOA of ≤ 0.65 in patients with a BMI < 30 and ≤ 0.55 in pts with a BMI ≥ 30 [20,26]. Although there is a clear inverse relationship between iEOA and aortic mean gradients, this cut-off value may have minimal clinical significance [26]. Rather than using iEOA values outlined in the VARC-3, some studies will define PPM as a mean aortic valve gradient of ≥ 20 mmHg calculated using echocardiography [15,26]. This is consistent with the mean aortic gradients used in the VARC-3 definition of moderate or severe HVD [20]. Rates of severe PPM have been shown to be higher in patients who undergo SAVR as compared to those who undergo TAVR [28,29,30]. Severe PPM after SAVR has led to higher mortality rates and heart failure hospitalizations; however, the evidence related to outcomes of severe PPM after TAVR remains limited [28,29,30]. Schofer et al., used VARC-2 definitions to determine moderate and severe PPM in a study of 1309 post-TAVR patients [31]. In this study, moderate and severe PPM occurred at rates of 22.9% and 12.9%, respectively [31]. Patients with an EF < 40% and severe PPM had significantly higher three-year mortality rates compared to those without PPM (45.1% vs. 68.0%, *p* = 0.041) [31]. In patients with an EF ≥ 40% and severe PPM, there was no significant difference in three-year mortality rate as compared to patients without PPM (29.5% vs. 34.6%, *p* = 0.96) [31]. 

Currently, self-expanding supra-annular valves are the preferred THV platform to reduce the risk of PPM in TAVR patients. Several studies have shown a lower incidence of moderate and severe PPM in self-expanding valves as compared to balloon-expanding valves [32,33,34,35,36]. In a large study comparing THV designs, patients who underwent TAVR with Evolut (self-expanding supra-annular valve) and Portico (self-expanding intra-annular valve) valves had similar increases in mean pressure gradients (7 mmHg) at 30 days post-TAVR despite the difference in positioning [35]. Both valves performed better when compared to the Sapien 3 balloon-expandable intra-annular valve (12 mmHg increase in mean pressure gradient at 30 days post-TAVR) [35]. However, in patients with small and very small native annulus, rates of moderate PPM were significantly higher with the Portico intra-annular valves as compared to the Evolut supra-annular valves [36]. In another study, intra-annular devices resulted in higher rates of moderate PPM (17.7% vs. 8.9%, *p* < 0.05) and severe PPM (1.6% vs. 0%, *p* < 0.05) compared to supra-annular valves, but these data are from older THV models and ultimately had no impact on 10-year survival [33]. 

## 2. Outcomes 

### 2.1. ViV TAVR vs. Redo-SAVR

The most recent ACC/AHA guidelines recommend that patients with severe symptomatic stenosis of a THV, bioprosthetic, or mechanical valve undergo redo surgical replacement unless the surgical risk is high or prohibitive [16]. However, recent observational studies have demonstrated that ViV TAVR may have better safety and efficacy than surgical replacement (redo-SAVR) of a THV or surgical bioprosthesis in patients with low-surgical risk as well [37,38,39]. Although there are no RCTs examining the long-term outcomes of ViV TAVR versus redo-SAVR, several meta-analyses of observational studies have been conducted to compare the short- and long-term outcomes of these procedures. Many of these studies demonstrated a reduction in 30-day mortality in patients who undergo ViV TAVR compared to redo-SAVR, but data regarding long-term mortality outcomes remain limited [11,37,38,39,40,41]. In one meta-analysis, 12 studies were included to compare the results of ViV TAVR to redo-SAVR procedures in patients with failed THVs or surgical bioprosthetic valves [37]. This study identified a significantly reduced 30-day mortality in patients receiving ViV TAVR in both unmatched (Odds Ratio [OR]: 0.52, *p* < 0.001) and matched (OR: 0.419, *p* = 0.003) populations [37]. However, this study also showed a significantly increased risk of PPM (OR 4.63, *p* < 0.001) in patients who underwent ViV TAVR compared to redo-SAVR [37]. The largest propensity-matched observational trial to date was conducted by Hirji et al., which showed reduced 30-day mortality (2.8% vs. 5.0%, *p* = 0.018), reduced major bleeding (35.8% vs. 49.9%, *p* < 0.001), and reduced length of hospital stay (7 vs. 9 days, *p* < 0.001) in patients who underwent ViV TAVR versus redo-SAVR [38]. The study showed no significant difference in the incidence of stroke, acute renal dysfunction, or need for permanent pacemaker placement between both groups [38]. Another meta-analysis of 15 observational studies found reduced 30-day mortality and acute kidney injury in ViV TAVR patients compared to SAVR patients (2.8% vs. 5.0%, *p* = 0.02) [40]. This study found that midterm mortality (1–2 years post-procedure) was similar between the two groups, but the incidence of severe PPM, prosthetic aortic regurgitation, and mean transvalvular gradient was higher in the ViV TAVR group [40]. The incidence of stroke and myocardial infarction and the need for pacemaker placement were similar between the two groups [40]. In another meta-analysis of 9 studies, similar findings regarding mortality, severe PPM, and mean transvalvular gradient were observed [41]. 

Although short-term safety and efficacy may favor ViV TAVR in high-risk patients, long-term durability and mortality differences between ViV TAVR and redo-SAVR remain unclear. In a meta-analysis including 12 observational studies (*n* = 3547), redo-SAVR showed a higher incidence of all-cause mortality within 30 days of the procedure when compared to ViV TAVR [42]. However, at the 1-year to 5-year time mark, redo-SAVR was associated with a lower incidence of all-cause mortality and cardiovascular death [42]. A major limitation of this study is the difference in baseline patient characteristics, as patients in the ViV TAVR group had a higher mean age and increased burden of comorbidities including diabetes, stroke, chronic obstructive pulmonary disease, peripheral vascular disease, atrial fibrillation, and history of coronary artery bypass. However, in a subgroup analysis containing 6 propensity-matched studies, redo-SAVR demonstrated lower mortality over the one- to five-year follow-up compared to ViV TAVR (Hazard Ratio 0.59, CI 0.44–0.79, *p* < 0.0001) [42]. 

Tam et al. utilized propensity score matching in their observational study to compare ViV TAVR and redo-SAVR [39]. The study population in this study was much smaller (*n* = 262), but the outcomes were more favorable for ViV TAVR with less 30-day mortality and improved 5-year mortality [39]. At 30 days, a 7.5% absolute risk reduction in mortality in the ViV TAVR group versus the redo-SAVR group was demonstrated [39]. At 5 years, the survival was significantly higher in the ViV TAVR group compared to the redo-SAVR group (76.8% vs. 66.8%, *p* = 0.046) [39]. ViV TAVR patients also had significantly reduced post-operation hospitalization time, need for blood transfusion, and need for pacemaker placement [39]. 

These studies are helpful in finding trends related to ViV TAVR vs. redo-SAVR, but multiple limitations exist, including small sample size, varying follow-up periods, absence of randomization, and significant differences in the burden of comorbidities between both groups, leading to selection bias. Since ViV TAVR is currently only approved for high-risk surgical patients, some ViV TAVR groups within these studies contained higher rates of patients with advanced age and multiple comorbidities that would place them at a higher likelihood of 5-year mortality regardless of the procedure performed. Many of these studies directly compare TAV-in-SAV to redo-SAVR, but some also include TAV-in-TAV patients within the ViV TAVR group which may also affect the results. In order to best compare the outcomes of patients undergoing ViV TAVR vs. redo-SAVR, RCTs are essential. Recent RCTs comparing ViV TAVR to native valve (NV) TAVR alongside results from the observational studies discussed above may help lead to the approval of ViV TAVR in low-risk patients. If approval were to be granted, the inclusion of low-risk surgical patients would allow RCTs with a large study population comparing TAV-in-SAV and redo-SAVR to be more feasible and the results more generalizable. 

### 2.2. ViV TAVR vs. Native Valve TAVR

Recent RCTs have shown similar or improved outcomes in low-risk surgical patients who undergo ViV TAVR (either TAV-in-SAV or TAV-in-TAV) compared to native valve (NV) TAVR [15,41,43]. In a large cohort of patients from the STS/CCC/TVT registry, clinical outcomes of NV TAVR and ViV TAVR were compared between low-, intermediate-, and high-risk groups [15]. The mortality rates at the 1-year follow-up were lower in the ViV TAVR patients in all risk groups when propensity matched against native TAVR recipients [15,43]. In addition to improved one-year mortality, ViV TAVR is also associated with lower rates of paravalvular leak, stroke, and need for permanent pacemaker placement when compared to NV TAVR [15,44]. However, with ViV TAVR, an increased risk of coronary obstruction, prosthesis–patient mismatch, and elevated transvalvular gradients has been described in the literature [15,43,44]. 

In a study using the STS/ACC TVT Registry in 2018, patients who underwent ViV TAVR had lower 30-day and 1-year mortality rates, decreased rates of stroke, and fewer heart failure hospitalizations when compared to patients who received NV TAVR [45]. In another study by Kaneko et al., 1-year mortality and new pacemaker placement were lower in the VIV TAVR group versus the NV TAVR group [15]. However, coronary artery obstruction was higher in the ViV TAVR group and occurred in 1.0% of ViV TAVR procedures across all risk groups [15]. In both studies, elevated post-procedure aortic valve gradients occurred in the ViV TAVR recipients. This resulted in increased reintervention rates but, ultimately, had no effect on mortality outcomes [15,45]. The PARTNER 2 trial found that high-risk surgical patients requiring a redo aortic valve replacement had better 5-year mortality outcomes as compared with high-risk patients receiving NV TAVR (50.6% vs. 73.0%, *p* < 0.0001) [46]. Long-term outcomes and durability data of ViV TAVR in low-risk and intermediate-risk patients remain scarce, and further studies are needed. 

### 2.3. TAV-in-TAV vs. TAV-in-SAV

Many studies use the term ViV TAVR to describe outcomes of patients who undergo TAVR of either a degenerated THV or surgical bioprosthesis. Landes et al. directly compared outcomes of patients who underwent TAV-in-TAV versus TAV-in-SAV and found that patients with a prior THV had increased procedural success compared to those with a surgical bioprosthesis (72.7% vs. 62.4%, *p* = 0.045) [47]. Success was determined by several factors including 30-day mortality, 1-year mortality, freedom from an additional intervention related to the device or major complication, mean gradient < 20 mmHg, and less than moderate AR. Notably, there were no significant differences in mean gradient ≥20 mmHg, safety, or mortality when TAV-in-TAV and TAV-in-SAV were directly compared [47]. However, despite larger aortic valve areas (1.55 cm^2^ vs. 1.37 cm^2^, *p* = 0.040) and mean residual gradients (12.6 mmHg vs. 14.9 mmHg, *p* = 0.011), TAV-in-TAV patients more often experienced mild AR at 30 days (36.1% vs. 17.2%, *p* = 0.003) [47]. These findings remained similar and statistically significant at 1 year [47]. Although more data is needed to better compare TAV-in-TAV versus TAV-in-SAV, some techniques may improve outcomes in patients who undergo SAVR and eventually require TAV-in-SAV.

Annular enlargement is one technique used during SAVR to reduce PPM and may facilitate future TAV-in-SAV procedures [48,49]. Different techniques (most commonly Nicks, Manouguian, and Konno) may be utilized to enlarge the aortic valve annulus by incising different structures including the mitral valve, interventricular fibrous trigone, and left atrial wall [48,49]. The use of annular enlargement has resulted in a reduction in PPM but historically has increased the perioperative mortality rate [49]. More recent studies have shown no increase in mortality with annular enlargement versus no annular enlargement at both 30 days (13% vs. 11% *p* = 0.55) and 5 years (11.3% vs. 9.1%, *p* = 0.46) [48]. These newer findings are likely related to increasing surgical experience with these techniques. Annular enlargement allows for increased valve sizing during SAVR and could potentially reduce PPM and mortality in patients who require future TAV-in-SAV. 

### 2.4. Procedural Planning in ViV TAVR

#### 2.4.1. PPM Risk Reduction

Mean transvalvular gradient ≥ 20 mmHg is one indicator of PPM that may occur in VIV TAVR due to constraints by the prior bioprosthetic valve, though VARC-3 definitions use iEOA to define PPM and its severity [15,20,45,50]. One-year mortality after TAV-in-SAV has been shown to be significantly higher in patients with smaller surgical valves, and it was thought that this may be due to resulting PPM [15,44,50,51]. To reduce the rates of PPM in patients with failed surgical bioprosthesis who undergo TAV-in-SAV, different techniques have been established and described in the literature, including bioprosthetic valve fracturing. 

Bioprosthetic valve fracturing was introduced to improve the expansion of the THV or allow placement of a larger THV ring by using high-pressure inflation of a non-compliant balloon to stretch or fracture the surgical valve ring [21,50,52]. Valve fracturing may be performed before or after the placement of the THV, and each timing has its advantages and disadvantages [21,50,52]. Fracturing before implantation of the valve allows for the confirmation of successful fracture before finalizing the size of the valve, while also making it easier to implant a self-expanding valve with less sizing mismatch [21]. However, with the use of pre-implantation fracture, there is an elevated likelihood of severe acute AR, posing a potential risk of hemodynamic instability [21]. Post-implantation fracturing allows for the most optimal valve expansion with balloon-expandable valves while also decreasing the risk of severe aortic regurgitation [21,50]. However, there is an increased risk of valve failure due to leaflet injury and migration and/or embolization of the valve [21,50]. Sathananthan et al. found that bioprosthetic valve fracturing after ViV TAVR was associated with improved valve expansion and lower gradients when tested ex vivo [52]. This, in addition to the reduced risk of acute hemodynamic instability due to severe AR, makes post-implantation fracturing more appealing. However, due to the lack of RCTs and evidence of long-term durability with post-implantation fracture, it remains unclear whether post-implantation fracture leads to better outcomes. 

Although PPM and elevated gradients are more common in ViV TAVR, the resulting elevated gradients may not necessarily have clinical consequences. The study by Kaneko et al. found higher rates of severe PPM (described as mean gradients ≥ 20 mmHg, *p* < 0.0001) in ViV TAVR versus NV TAVR, but the patients with mean gradients ≥ 20 mmHg had similar 1-year survival compared to those with gradients < 20 mmHg (9.8% versus 9.9%, *p* = 0.674) [15]. Chhatriwalla et al. found that bioprosthetic valve fracture was associated with increased aortic valve area and lower mean gradient, but often resulted in higher in-hospital mortality and similar 1-year mortality [53]. Together, these findings demonstrate that fracturing may be successful in improving hemodynamics but may not necessarily lead to improved outcomes. Thus, future studies are indicated to determine the long-term outcomes of various ViV TAVR gradients and the long-term risks and benefits of fracturing to improve those gradients. 

As discussed earlier, the use of supra-annular devices may reduce the likelihood of PPM in NV TAVR. This is also likely to be true for ViV TAVR, though current data are lacking. In addition to the potential lower rates of PPM, supra-annular THVs may also reduce the risk of thrombosis in ViV TAVR. In vitro, the confinement of THV leaflets during ViV intra-annular positioning leads to increased blood stasis and may result in leaflet thrombosis [54]. While supra-annular THVs may reduce the risk of PPM and thrombosis, the location of the implant decreases the mean valve to coronary distance (VTC), resulting in an increased likelihood of coronary obstruction [54,55,56]. 

#### 2.4.2. Coronary Protection

When performing TAVR, the risk of coronary obstruction is very low; however, the rate of coronary obstruction and acute coronary syndrome (ACS) is higher in patients undergoing ViV TAVR as compared to NV TAVR [15,18,57]. Coronary obstruction in TAVR can be divided into three categories, namely acute, early-delayed, and late-delayed [55,58]. Acute coronary obstruction is most often related to the extension of surgical bioprosthetic valves or THV leaflets in close contact with a coronary ostium [18,21]. After implantation of the new THV, the bioprosthetic valve leaflets are displaced in an upward fashion, which may lead to the obstruction of a coronary artery [18,21]. Acute coronary obstruction is a complication associated with a high mortality rate and may occur in 2.3–3.5% of patients who undergo ViV TAVR [18,58,59,60]. Delayed coronary obstruction is a less commonly reported complication of TAVR that may affect a significant number of patients. When compared to NV TAVR, ViV TAVR patients were > 4 times more likely to have delayed coronary obstruction (0.89% vs. 0.18%, *p* < 0.001) [55]. A majority of these events occurred within 7 days of ViV TAVR (an early-delayed event) and were likely to be related to the displacement of a calcified native valve leaflet, a displaced surgical valve leaflet, or continued expansion of the valve [55]. When coronary obstruction occurs beyond 7 days (a late-delayed event), bioprosthetic endothelialization and partial obstruction by a native or bioprosthetic leaflet are the presumed predominant mechanisms [55]. 

Predictors of coronary obstruction in ViV TAVR are dependent on both the degenerated THV or bioprosthetic valve and the native aortic anatomy [61]. Narrow sinotubular junction, narrow sinuses of Valsalva, low coronary ostia, and previous aortic root surgery are all examples of aortic anatomy that may increase the risk of coronary obstruction [61,62]. Bioprosthetic surgical valves with externally mounted leaflets are more likely to cause coronary obstruction during TAV-in-SAV due to the close proximity of the leaflets to the coronary arteries [61]. Stentless bioprosthetic valves and valves with bulky or outward-extending leaflets may also increase the likelihood of coronary obstruction [61,62]. When a THV is implanted during ViV TAVR, bioprosthetic leaflets from the prior SAV are tilted upward, creating a tunnel of tissue often described as a neo-skirt that could potentially lead to coronary obstruction that restricts future coronary access [61,63]. During TAV-in-TAV placement, intra-annular leaflet positioning may be preferable for reduced coronary obstruction and better coronary re-access, but there are no clear data trends established to determine if this benefit outweighs the risk of PPM and paravalvular leak [61,62,63]. 

In order to prevent coronary obstruction related to ViV TAVR, risk assessment is crucial. A multidetector computed tomography scan should be performed to measure VTC ostia distances. Any distance < 3 mm is considered high risk for coronary obstruction, while a distance of 3–6 mm indicates intermediate risk and ≥6 mm indicates low risk [18,21]. Aortic root and coronary angiography may also be beneficial in the evaluation of the patient’s anatomy and the positioning of the prior bioprosthetic valve [18]. For those at elevated risk, different techniques have been introduced in an attempt to limit coronary obstruction and in-hospital mortality related to coronary obstruction in ViV TAVR. 

The placement of undeployed coronary balloons/stents with the help of guide catheter extensions into the coronary artery at risk before TAVR valve implantation can be performed using the chimney (or snorkel) technique [21,59,64,65,66]. The chimney technique has also proven to be an acceptable bailout technique to re-establish flow through the obstructed coronary artery if an acute coronary obstruction occurs during the TAVR procedure [65,66]. Specific orientation and valve commissure alignment during the deployment of a THV are additional techniques to lower the risk of significant coronary obstruction, though with older generation THVs, this was difficult to achieve [57,67]. 

The bioprosthetic aortic scallop intentional laceration to prevent iatrogenic coronary obstruction (BASILICA) procedure was introduced to provide a safer option for patients who are at increased risk of coronary obstruction [21,59,60,66,68]. BASILICA is a technique that uses standard cardiac catheterization equipment to deliver radiofrequency energy to native or bioprosthetic aortic valve leaflets to split the leaflet [59]. Once a THV is implanted, the native or prior bioprosthetic valve leaflets will still be displaced upwards, but the split leaflets splay away from the coronary ostia, reducing the risk of coronary obstruction [59,60]. In the initial BASILICA trial, 30 high-risk subjects with a VTC distance of <4 mm were enrolled. 100% of these patients experienced freedom from coronary obstruction with the use of the BASILICA technique [59]. Notably, there was a 10% stroke incidence in these patients, but given the small sample size (n = 30) it is unclear if the BASILICA technique increases the risk of stroke [59]. Further studies with a larger population are needed to determine the safety and efficacy of the BASILICA technique in patients at high risk of coronary obstruction. More recently, the ShortCut dedicated leaflet-splitting device has shown success in both bench testing and on eight human subjects [69]. This device may provide a new way to split bioprosthetic valve leaflets and prevent coronary obstruction, though larger studies regarding its safety and efficacy are still required. 

Due to an increased risk of coronary artery obstruction in patients who undergo ViV TAVR, the techniques discussed in addition to new THV technologies are vital to reducing the risk of acute coronary obstruction and preserving coronary access. Commissural alignment is critical for the optimization of THV placement, and by positioning the commissural posts of the THV away from the coronary ostia, the risk of coronary obstruction can be reduced [66,67]. The orientation, size, shape, depth of valve insertion, and force applied during insertion must all be taken into careful consideration to ensure proper commissural alignment during the deployment of a THV [66,67]. Newer generation self-expanding THVs contain specific markers to help guide operators to perform correct commissural alignment [66,67]. Each new generation self-expanding THV has slightly differing steps and markers associated with commissural alignment, but all have demonstrated a more optimal commissural alignment leading to reduction in coronary obstruction and the preservation of coronary access [67]. 

## 3. Conclusions

ViV TAVR is a feasible procedure for aortic valve replacement in patients with failed THV and surgical bioprosthetic valves. As the trend toward the use of surgical bioprostheses continues to increase, the potential risks and benefits of ViV TAVR for patients in all surgical risk groups are becoming increasingly relevant. Studies thus far have demonstrated that ViV TAVR has similar and possibly improved mortality outcomes in high-risk patients when compared to NV TAVR and redo-SAVR. While much of the literature shows acceptable and improving short-term and long-term mortality rates, there is still a concern for an increased risk of PPM and coronary obstruction in patients who undergo ViV TAVR. Several adjustments to newer generation THVs and new techniques have been developed to decrease the rates of these complications, but they continue to be studied and perfected. The lack of approval for ViV TAVR in low-risk and intermediate-risk patients limits the ability to perform large-scale RCTs that study the short- and long-term efficacy of ViV TAVR compared to NV TAVR and redo-SAVR. Further randomized trials with a large study population over longer periods are needed to better understand the safety, efficacy, and durability of ViV TAVR in patients in any surgical risk group. 

## Abbreviations

AKI = acute kidney injury; BASILICA = bioprosthetic aortic scallop intentional laceration to prevent iatrogenic coronary obstruction; BSA = body surface area; HF = heart failure; PPM = prosthesis–patient mismatch; TAVR = transcatheter aortic valve replacement; THV = transcatheter heart valve; ViV = valve-in-valve.
